# State-of-the-Art Challenges and Perspectives in Multi-Organ Cancer Diagnosis via Deep Learning-Based Methods

**DOI:** 10.3390/cancers13215546

**Published:** 2021-11-04

**Authors:** Saqib Ali, Jianqiang Li, Yan Pei, Rooha Khurram, Khalil ur Rehman, Abdul Basit Rasool

**Affiliations:** 1 Faculty of Information Technology, Beijing University of Technology, Beijing 100124, China; alisaqib@emails.bjut.edu.cn (S.A.); lijianqiang@bjut.edu.cn (J.L.); rehmankhalilur@emails.bjut.edu.cn (K.u.R.); 2 Computer Science Division, University of Aizu, Aizuwakamatsu 965-8580, Japan; 3 Beijing Key Laboratory for Green Catalysis and Separation, Department of Chemistry and Chemical Engineering, Beijing University of Technology, Beijing 100124, China; khurramrooha@emails.bjut.edu.cn; 4 Research Institute for Microwave and Millimeter-Wave (RIMMS), National University of Sciences and Technology (NUST), Islamabad 44000, Pakistan; arasool.msee17seecs@seecs.nust.edu.pk

**Keywords:** cancer diagnosis, machine learning, deep learning, medical imaging, automated computer-aid diagnosis systems

## Abstract

**Simple Summary:**

Cancer is a deadly disease that needs to be diagnose at early stage to increase patient survival rate. Multi-organ (such as breast, brain, lung, and skin) cancer detection, segmentation and classification manually using medical imaging is time consuming and required high expertise. In this study, we summarize existing deep learning segmentation and classification methods for multi-organ cancer diagnosis and provide future challenges with possible solutions. This review may benefit researchers to design new robust approaches that could be useful for the medical specialists as a second view.

**Abstract:**

Thus far, the most common cause of death in the world is cancer. It consists of abnormally expanding areas that are threatening to human survival. Hence, the timely detection of cancer is important to expanding the survival rate of patients. In this survey, we analyze the state-of-the-art approaches for multi-organ cancer detection, segmentation, and classification. This article promptly reviews the present-day works in the breast, brain, lung, and skin cancer domain. Afterwards, we analytically compared the existing approaches to provide insight into the ongoing trends and future challenges. This review also provides an objective description of widely employed imaging techniques, imaging modality, gold standard database, and related literature on each cancer in 2016–2021. The main goal is to systematically examine the cancer diagnosis systems for multi-organs of the human body as mentioned. Our critical survey analysis reveals that greater than 70% of deep learning researchers attain promising results with CNN-based approaches for the early diagnosis of multi-organ cancer. This survey includes the extensive discussion part along with current research challenges, possible solutions, and prospects. This research will endow novice researchers with valuable information to deepen their knowledge and also provide the room to develop new robust computer-aid diagnosis systems, which assist health professionals in bridging the gap between rapid diagnosis and treatment planning for cancer patients.

## 1. Introduction

Cancer diagnosis using different medical images plays a significant role in detecting various abnormalities, for instance, skin cancer [1], breast cancer [2], lung cancer [3], brain tumors [4,5], blood cancer [6], and so forth. Tumor-induced abnormalities are the leading source of universal demise [7]. The GLOBOCAN 2020 report illustrates that lung cancer (18%) is the leading cause of death; other cancers are also life-threatening for humans with different mortality rates, for example, breast cancer (6.9%) and brain cancer (2.5%) [8]. Many image modalities are utilized to analyze irregularities in different organs, such as Magnetic Resonance Imaging (MRI) [9], Positron Emission Tomography (PET) [10], Computed Tomography (CT) [11], and mammography [12].

The human brain is the most complex part of our body. The functioning of brain cells is highly influenced by the irregular mitosis mechanism. As a result, cancer cells are produced with distinct morphological properties, such as size, shape, boundaries, and so forth. Low-grade gliomas (grades I and II) and high-grade glioma (grades III and IV) are two major categories of brain tumors. Low-grade tumors grow slowly [13], while the high-grade are the most malignant primary brain tumors, which are more aggressive and disrupt the blood–brain supply [14]. Glioblastoma (GBM) [15], a grade IV glioma, is the most common, invasive, and lethal type of primary brain tumor. Cancerous cells are less contrasted than the nearby cells, making perfect brain tumor recognition challenging. Thus far, examining MR scans is one of the most effective techniques for detecting brain tumors owing to its non-invasive nature, painless test procedure and for manipulating the tumorous region from various angles [16].

A leading cause of death among women is breast cancer. Abnormal cells can be benign or malignant. Malignant breast cancer is more aggressive and threatening because it spreads to multiple body organs via the lymphatic system [17]. A benign tumor, a noncancerous tumor, is well recognized in type and has a large size, but malignant tumors are diffused and small. Due to narrow size and fatty tissue problems, the early detection of malignant tumors is challenging. The timely detection of breast cancer can help with the diagnosis procedure, which can alleviate the disease severity with more excellent recovery [18,19]. Therefore, state-of-the-art, fully automatic methods are needed for early breast tumor detection.

The formation of a certain nodule in the lungs is an indication of lung cancer. A round-shaped nodule in the lungs can be benign or malignant [20]. The malignant nodules develop swiftly, and their rapid progression might affect the other body parts. CT images are a commonly employed diagnostic technique for lung cancer detection [21].

**Figure 1:**
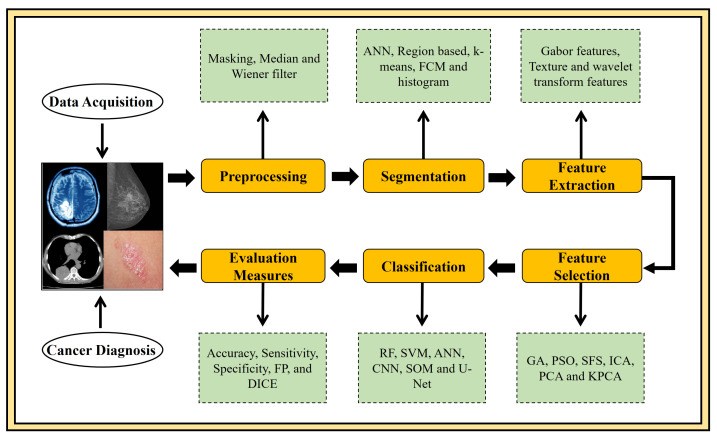
A flowchart of a standard CAD system for diagnosing multi-organ of human body cancer. This flowchart illustrates all steps of a CAD system.

In addition to the above-mentioned types of cancer, skin cancer is a fast dominant disease worldwide [22]. It has two categories, melanoma, and non-melanoma cancer. Melanoma cancer is considered aggressive than non-melanoma, a critical type of cancer that arises as a dark spot on the skin. Occasionally, these spots grow as a mole to progress in shape, uneven edges, and different skin colours. In the last year (2020), the death count for non-melanoma and melanoma skin cancer is 63,731 and 57,043, respectively [23,24]. To summarize, to cope with a fatal disease like cancer, early diagnosis is highly needed to guard the patients’ life, which could be done only by developing advanced CAD systems. Computer-aided procedures currently play an important role in medical image analysis. The CAD-aided segmentation and classification facilitate target separation, diagnosis, quantitative measurements, and treatment planning. Figure 1 depicts the commonly employed CAD approaches in the domain of medical image analysis. So far, many methods have been developed for various types of detection, segmentation, and classification. However, research in this area is still in its infancy. This study critically highlights the room for improvement in four major cancer types viz. lung, breast, brain, and skin in theoretical and technological ways. Moreover, this article covers cutting-edge methods for multi-organ cancer detection and diagnosis using medical images, which would be highly beneficial for novice researchers to propose the CAD system in a specific domain. Additionally, a comprehensive analysis of the most commonly used standard databases for the brain, breast, lung, and skin cancers is also elaborated. Majorly this review includes the brief discussion part where open research challenges and future directions are extensively discussed, which can assist physicians and radiologists in treatment planning.

The rest of the study is structured in several sections: Section 2 presents material and methods that include a brief explanation of the standard datasets available for multi-organ images and performance evaluation metrics. Section 3 contains the brain tumor detection methods. Section 4 is about breast cancer detection methods; Section 5 represents the lung cancer detection methods; Section 6 describes the skin lesion cancer detection methods. Section 7 is about the discussion and open research challenges along with the state-of-the-art solutions and prospects. In Section 8, the whole study is summarized under the conclusion heading.

The key motivation behind this conducted research is to look for the answer to these queries:

1. What is the commonly employed imaging modality in each of the four cancers?
2. What kind of databases is utilized for medical image analysis?
3. Which kind of AI technology is in trend for the early diagnosis of these cancers?
4. Why is CNN architecture is a trend in breast, brain, lung, and skin cancer diagnosis?
5. What performance evaluation metrics are employed to evaluate the models’ efficiency?

## 2. Material and Methods

In this section, we have briefly discussed search strategy and selection criteria. Moreover, some standard datasets are described, which are implemented extensively for cancer segmentation, and classification approaches followed by performance measures are also briefly explained.

### 2.1. Search Strategy and Selection Criteria

In this review, we have utilized openly available search databases such as Google Scholar and PubMed to find most related articles using different queries. We have limited our search to manuscripts published between the years 2016–2021. We have used the following queries in different combinations: “brain cancer diagnosis”, “Brats dataset segmentation”, “breast cancer diagnosis”, “lung cancer diagnosis”, “skin cancer diagnosis”, “LIDC/IDRI database segmentation and classification”, “skin lesions”, “brain tumor segmentation and classification”, “breast cancer segmentation and classification”, “WBCD dataset segmentation”, “lung cancer segmentation and classification”, “skin cancer segmentation and classification”, “PH2 dataset segmentation”, “brain tumor detection using machine learning and deep learning classifiers”, “breast tumor diagnosis using machine learning and deep learning classifiers”, “lung cancer detection using machine learning and deep learning classifiers”, “skin cancer diagnosis using machine learning and deep learning classifiers”, “brain tumor MRI and deep learning”, “DDSM classification”, “artificial intelligence and breast cancer”, “artificial intelligence and brain tumor”, “artificial intelligence and lung cancer”, and so forth. More than 300 related papers are reviewed, among them 156 papers are selected for the current study, and 111 manuscripts out of 156 were most relevant to brain, breast, lung, and skin cancer diseases.

### 2.2. Most Popular Publicly Available Datasets

#### 2.2.1. Multimodal Brain Tumor Image Segmentation Benchmark(BraTS) Database

BraTS dataset consisted of multi-institutional routine clinically acquired pre-operative multimodal MRI scans of High-Grade Glioma, that is, Glioblastoma (GBM/HGG) and Lower Grade Glioma (LGG), with a pathologically confirmed diagnosis and available overall survival (OS), are provided as the training, validation and testing data. In the MRI study, BraTS focuses on evaluating advanced techniques of brain tumors segmentation and classification. MRT multi-institutional pre-operative images are utilized to segment brain tumors, specifically gliomas that vary in appearance, shape, size, and histology. The patients’ OS is also brought into focus by integrative radiometric features analysis. It has eight popular dataset collections from 2012 to 2020. The datasets used in the BraTS challenge are acquired from 3T multi-modal scanners with ground truth annotated and confirmed expert board-certified neuroradiologists [25].

#### 2.2.2. Lung Image Database Consortium image collection(LIDC/IDRI) Database

The dataset, named LIDC and IDRI, are commonly employed datasets for implementing recognition about the lung nodule. LIDC-IDRI is a freely available databank of CT lung images. It contains scans of nodule outlines and subjective nodule properties rankings. It is established to help lung nodules study and comprises 1018 cases with 244,617 CT scans and XML report files generated by experienced thoracic radiologists [26].

#### 2.2.3. Digital Database for Screening Mammography(DDSM) Database

It is a standard reference of mammographic scans generated in Massachusetts General Hospital. Digital database for screening mammography contains 2620 cases, 1935 images composed of tumors. Each case has four-view mammography screenings images of breast and subject information. Suspicious areas and relevant pixel-level ground truth information annotated by the radiologist (normal, benign, and malignant image) are included in images [27].

#### 2.2.4. Wisconsin Breast Cancer Database(WBCD) Database

A commonly employed multivariate Wisconsin Breast Cancer Database contains 683 patients’ information from the biopsy test of female breast cancer. There are 11 attributes on each record. The first ten columns comprise the attributes column, and the 11th column holds class attributes. WBCD has 444 benign examples and 239 malignant examples for training and testing tasks [28].

#### 2.2.5. International Skin Imaging Collaboration(ISIC) Database

ISIC has a baseline dataset of 25,331 JPEG dermoscopic images for training and 8238 for testing skin lesions that are openly accessible. It describes outstanding problems in the segmentation and classification of skin lesions, incorporated with a high-resolution image validated by experienced experts. The dimensions of the scans are unbalanced because they use various types of visual sensors [29].

#### 2.2.6. PH2 Database

PH2 has been developed for the detection of skin lesion cancer. It is a dermoscopic image database acquired at the Dermatology Service of Hospital Pedro Hispano, Matosinhos, Portugal. The database contains 200 melanocytic skin lesion images for research purposes, in which 80 images are benign cancer, 40 images belong to malignant, and the remaining 80 images are associated with the suspicious lesion. These are 8-bit RGB color images with having dimensions of 768 × 560 pixels [30].

The summary of the above-mentioned and other related datasets for various cancer diseases for the example database name, modality, number of images or patients, link to the source, is presented in Table 1.

### 2.3. Performance Evaluation Metrics

The major evaluation measures to check the performance of segmentation and classification tasks of DL models such as Accuracy (Acc), Specificity (SP), Sensitivity (SN), area under the curve (AUC), and true positive (TP) are reviewed in this research. SN, also entitled as a recall, is a probability of recognizing the segmented image’s true pixels. SP depict the ability to recognize negative pixel. Acc is defined as the ratio among the correctly identified pixels (TP + TN) and the total pixel in an image (TP + FP + TN + FN). The mathematical formulas to compute these performance measures are given in Equations (1)–(4).

(1) $$SN=\frac{TP}{TP+FN}$$

(2) $$SP=\frac{TN}{TN+FP}$$

(3) $$Acc=\frac{TB+TN}{TP+FP+TN+FN}$$

(4) $$AUC=\frac{1}{2}\left(\frac{TP}{TP+FN}+\frac{TN}{TN+FP}\right)$$

## 3. Brain Tumor

A major contributing factor towards the universal death rate is a brain tumor. The World Health Organization (WHO) broadly categorized brain tumors as benign and malignant tumors as from Grades I–IV (lowest–extreme aggressive) [31]. Grades I and II belong to the Low-grade (LG) tumors category and are also termed benign tumors. Grade III and IV belong to the High-grade (HG) tumors category and are also termed as malignant tumors [32]. LG and HG tumors also differ in terms of their growth and the number of years of life anticipation. Therefore, timely diagnosis of brain tumor is necessary to minimize the universal death rate. However, distinguishing healthy tissue from tumor is not an easy task, owing to varying shapes and sizes, poor contrast and variable locations [31]. These factors influence the complexity of tumor growth and predict the extent of resection at the time of surgical planning, this has repercussions in terms of patient management [33]. Therefore, reliable tumor classification and segmentation are important tasks for the determination of tumor size, exact position, and type. For that purpose, computed tomography (CT), biopsy, cerebral angiography, positron emission tomography (PET) and MRI are significant medical imaging modalities [34]. Among them, MRI scans have attracted greater attention owing to their non-invasive nature and their in-depth as well as objective analysis. MRI is a potent and sensitive modality for detecting brain tumors and their boundary delineation [35]. Initially, the precise analysis and comprehensive monitoring of brain tumors were dependent on the radiologist experts. However, the radiologist’s dependent process is tedious and time-consuming. Therefore, the development of advanced CAD systems truly helps radiologists for the improved and timely diagnosis of brain tumor. Many articles have been published on brain tumor detection, classification, and segmentation to date. Some researchers apply conventional ML-based feature extraction methods for brain tumor detection.

Nilesh Bhaskarrao et al. [36] proposed a brain tumor segmentation technique by applying Berkeley wavelet transform (BWT) with support vector machine SVM. BWT was used for the feature extraction task, followed by the SVM classifier to perform the classification task. The authors reveal that the results obtained 96.51%, 94.2%, and 97.72% for accuracy, specificity, and sensitivity. Alfonse et al. [37] used the SVM method for automated brain tumor segmentation and classification using MR images. Firstly, brain images are segmented, employing adaptive thresholding. Secondly, features are extracted using Fast Fourier Transform (FFT), then Minimal Redundancy Maximal Relevance methods are used for feature selection. This technique achieved 98.9% classification accuracy. In SVM, the classification of different points based on proximity accompanied by splitting hyper-plane required more execution time to calculate linear or quadratic complications. Wu et al. [38] introduced a multi-level Gabor wavelet method to reduce the linear or quadratic calculations by using image superpixels instead of image voxels for the segmentation of GBM. Extracted features are fused to SVM for classification purposes. Recently, an effort reducing the classification error using SVM is presented by Soltaninejad et al. [39] proposed to classify tumor grades (for example, II, III, and IV) using statistical features extraction. ROI was segmented manually or by a superpixel-based method. The better version of brain tumor grading for MRI image analysis is developed in [40].

Tianbao Ren et al. [41] developed an automated brain tumor segmentation approach. Initially, the authors used histogram equalization to acquire the related information. Then they implemented an improved Kernel-based Fuzzy C mean (KFCOM) with Weighted fuzzy kernel clustering (WKFCOM) model that enhances brain image segmentation performance. The results illustrate that the proposed combined algorithm achieves an improved misclassification rate which was less than 2.36%. In comparison to FCM methods, a state-of-the-art technique [42] is introduced that categorized White Matter, Grey Matter, and cerebrospinal fluid spaces using Adaptive Fuzzy K-mean (AFKM) Clustering. Researchers declare that the implemented AFKM algorithm obtains superior results in contrast to FCM both qualitatively and quantitatively.

Li et al. [43] suggested a multi-modality deep learning network for brain tumor segmentation that extracts multi-scale features of brain tissue from the MR scans. A data mining system via the combination of FCM and SVM was presented for the MR image segmentation in [44]. A combined technique with k-mean and FCM was introduced [45]. This technique implemented a median filter for MR brain images denoising and a brain surface extractor for features extraction. Then, clustering is done through the proposed method. Anitha Vishnuvarthanan et al. [46] suggested a hybrid algorithm that involves Bacteria Foraging Optimization (BFO) and a Modified FKM clustering approach for the segmentation and classification of brain MR images.

**Figure 2:**
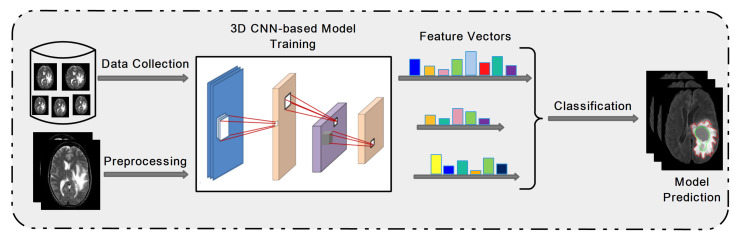
Overview of 3D CNN architecture for brain tumor prediction.

Deep learning is an emerging approach and extensively applied in many object detection applications to automatically perform feature extraction for complex patterns. The most well-known method is a convolutional neural network (CNN); an example of a 3D architecture for brain tumor detection is presented in Figure 2. Multiple hidden layers of CNN with batch normalization architecture was implemented for brain tumor classification [47].

The developed classification model was evaluated on BRATS 2013 and achieved 0.99% accuracy. Recently transfer learning approach is implemented in several medical domains. It employs pre-trained networks that are previously trained on massive datasets like ImageNet. Resent 50, GoogleNet, and VGG 19, are examples of pre-trained learning networks broadly used to resolve classification issues [48]. S. Deepak and P.M. Ameer [49] developed a CNN-based GoogleNet transfer learning classification model to classify brain tumors, including glioma, meningioma, and pituitary. The proposed algorithm attained better accuracy 92.3%, which was more enhanced to 97.8% by applying multiclass SVM. To improve the CNN-based model performance in terms of accuracy, researchers suggested a combined framework using Stationary Wavelet Transform (SWT) and Growing Convolution Neural Network (GCNN) for brain tumor segmentation [50]. SWT technique was applied for feature extraction rather than Fourier transform that provides improved results for discontinuous data followed by the Random Forest method for the classification task. The suggested technique contributes a 2% improvement compared with traditional CNN. The in-depth features are learned by applying transfer learning models like AlexNet and computing the scores of each feature matrix. The obtained feature vector scores are merged to generate a final feature vector. The combined features vector is given to the classifier to examine the performance of the proposed method [51]. The hand-crafted and machine-learned-based features fusion like semantic segmentation network (SegNet) is also used to segment brain tumors [52]. A summary of brain tumor cancer diagnosis CAD systems is illustrated in Table 2.

The systematic literature analysis showed that deep learning technology resulted in great realistic performances in brain tumor image analysis. It has been observed that the most commonly employed ML method is SVM. However, deep learning algorithms are the top performers, especially DCNN. However, the main limitation of DCNN is a dependency on massive training data with expert radiologists’ annotations from different institutions. It is a pretty tricky task. We believe the hybrid intelligent systems designed by integrating machine learning approaches with other methodologies like deeply learned approaches offer a highly proficient, accurate classification system. It appears to give higher classification accuracy in the range of 95–100%. Moreover, CNN can be known as an archetypical classifier owing to immense usage in the prognosis of various diseases such as brain tumor classification, segmentation, and detection. Most of the publications were exploited BRATS data sets for tumor diagnosis tasks.

## 4. Breast Cancer

Several image processing-based architectures in collaboration with artificial intelligence and ML are reported for the performance enhancement of medical detection and diagnostics processes. CAD systems are considered as a robust approach in the modern diagnosis and detection of breast cancers using medical imaging [68]. Robust CAD systems increase the characteristic of images and improve the diagnostic capability of healthcare professionals. The researchers apply five imaging modalities such as ultrasound, MRI, mammography, thermography, histology, and so forth, for breast cancer diagnosis. Generally, the human breast is observed as a supersensitive organ of the body; thus, few of these discussed medical modalities are suggested. Across all modalities, mammography is recommended because it is a reliable way to detect breast cancer in the early stages. Eight benchmark imaging databases of the breast exist and are freely available on the internet for breast cancer diagnosis, termed INbreast, Mammographic Image Analysis Society (MIAS), Wisconsin Breast Cancer Dataset (WBCD), Image Retrieval in Medical Applications (IRMA), Database for Screening Mammography (DDSM), Wisconsin Diagnosis Breast Cancer (WDBC), breast cancer data repository (BCDR), and Breast Cancer Histopathological Image (BreakHis) [69]. During pre-processing some necessary operations are applied to better image quality, like contrast improvement, noise reduction, and artifact removal.

After the preprocessing, breast mass segmentation is the next crucial stage for increasing the accuracy of detection systems with decreasing false results about existence of abnormality [70]. Segmentation of breast tumor scans is challenging due to numerous obstacles like rough or lobulated corners, breast lumps or tissues, pectoral muscle, and mutual values of mass intensities. These problems complicate the procedure of diagnosis systems in facilitating the health care experts. Usually, segmentation provides localization of breast tumors or lesions and detection within two-dimensional or three-dimensional images. A prominent feature of breast cancer is silhouette and contour, as they provide essential information about the metastatic nature of the breast mass.

**Figure 3:**
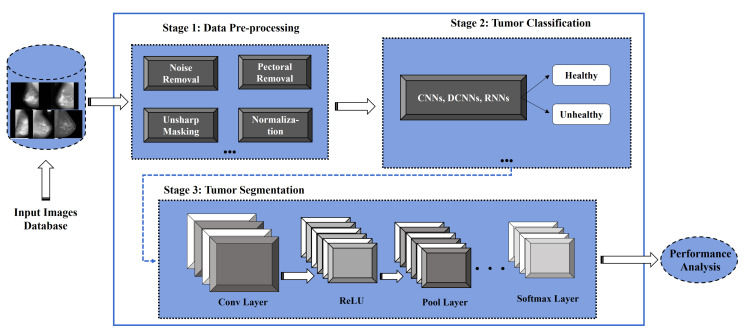
DL-based framework for breast tumor classification and segmentation.

From the literature survey, segmentation methods of breast mass are classified into color-based [71], contour-based, morphological-based, threshold-oriented-based [72], region-oriented, DL-based network [73,74], and hybrid segmentation methods [75]. At present, DL-based applications developed via CNN are gaining more attention for breast cancer detection and segmentation through CAD systems [74,76], as presented in Figure 3.

Breast cancer prognosis is highly dependent on the classification results of morphological samples and cell surface receptors including hormone receptor status [77]. Breast cancer classification is generally done by three methods: supervised classification, unsupervised classification, and semi-supervised classification. The perfect classification performance of morphological data depends on extracting important features such as statistical, shape, and textural [78]. These extracted features are forwarded to different ML/DL classification algorithms for training, validation, and testing model. Neural networks (NN) [79], Support vector machines (SVM) [80], and k-nearest-neighbor (KNN) [81] are few robust and powerful computational algorithms that are helpful for complex classification challenges.The use of AI systems is an additional development in the medical imaging domain. In particular, the practice of CNN-based classification algorithms and hybrid frameworks are adopted that have produced encouraging outcomes in breast cancer detection. The complete summary of current approaches is illustrated in Table 3.

The following observations are made from the aforementioned literature analysis: (1) most popular utilized classifiers are deep learning, SVM, CNN, and KNN; (2) Mammogram is the widely employed modality for breast cancer diagnosis; (3) For the forthcoming robust design of CAD schemes, the development of 3D mammogram-based CAD schemes could be the new trend; (4) For the effective and in-time diagnosis of breast cancer instead of sole mammogram, other modalities like CT, ultrasound, thermal and histological images must be considered. Boundary images should be labeled for the classification of multi-class breast cancer because they allow researchers to analyze the usefulness of the recently established multiple classes breast cancer model.

## 5. Lung Cancer

**Figure 4:**
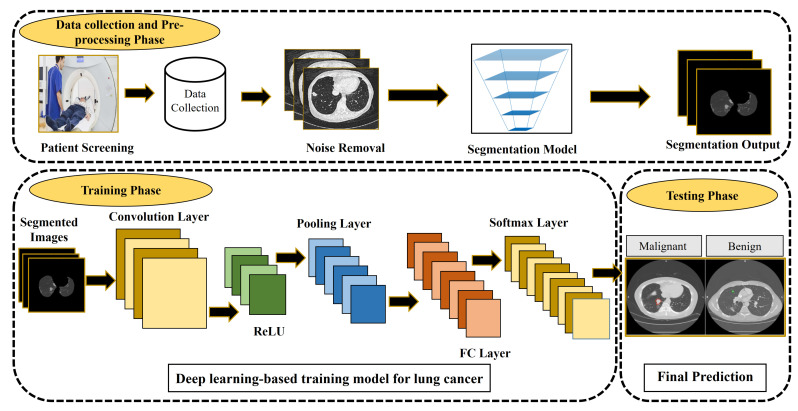
CAD system for lung cancer detection.

Advanced CAD systems help in timely lung cancer diagnosis and/or prognosis by working on the CT images using AI approaches. These CAD-based decision support systems examine the input CT scans, employing various methods to segment and classify medical images as presented in Figure 4. Various databases have been employed so far for lung cancer diagnosis for instance Automated Nodule Detection Database (ANODE09), the Early Lung Cancer Action Program (ELCAP) database, The LUNA16 dataset, the Lung Image Database Consortium, and Image Database Resource Initiative (LIDC-IDRI), and so forth. Among them, LIDC-IDRI is used as the gold standard database for the evaluation of lung cancer detection techniques [89]. The polygon approximation technique is proposed to detect nodule shape properties [90]. The intensity and geometric features vector then fused to the SVM classifier to identify actual nodules. The proposed algorithm evaluated on the LIDC dataset achieved promising results of 98.8%, 97.7%, 96.2% in terms of accuracy, sensitivity, and specificity. Currently, 3D-based segmentation is a robust method to accurately detect nodules from lung images. Many approaches have integrated these characteristics. Paing et al. [91] developed a fully automated and optimized random forest approach to classifying pulmonary nodules using tomography scans. A 3D chain code algorithm is applied to improve the borders. The public dataset contains 888 images evaluated on the proposed algorithm that obtained satisfactory results of 93.11%, 94.86%, and 91.37% accuracy, specificity, and sensitivity. The false positives per scan were only 0.086.

Deep learning makes breakthroughs owing to the latest developments in image processing, particularly medical image examination. The attention of CAD systems has shifted from traditional hand-craft features to automated deep-learned features. Its performance is superior in terms of segmentation and classification of nodular items through CT scan images. Perez, G., and Arbelaez, P. proposed a 3D CNN approach for the diagnosis of lung cancer automatically and obtained effective results for recall of 99.6% and AUC of 0.913% [92]. The model was trained on the LIDC-IDRI standard dataset to evaluate the performance. In DL, autoencoder and softmax are well-known and suitable methods for feature selection and classification. Apart from this, a different variant of the 3D CNN method is also producing superior results for extracting features automatically [93]. CNN architecture contains several hidden layers such as convolution, max-pooling, and a fully connected layer with a softmax function for image classification [94,95]. In recent years, DL-based CAD algorithms are developed, for example, 3D U-Net [96], patch-based 3D U-Net [97], and hybrid CNN algorithm [98]. Moreover, integrating ML and DL techniques also enhance the performance for segmentation as well as classification [99]. Table 4 shows existing literature on different method’s performance for lung nodules detection.

From the literature analysis of lung cancer diagnosis, it was found that the CT scan is a widely employed modality in the CAD system. The researchers believe that 3D CNNs would provide more promising results than 2D CNN owing to the ability of the model to manipulate spatial information via 3D convolution and pooling operation. Up till now few works is reported in literature where 3D CNNs are employed in medical image analysis for lung cancer diagnosis [101,105,113,114]. Overall, some reports exhibit good performances in segmentation and classification. However, still, some methods have some limitations like low sensitivity, high FP, and more time consumption. Therefore, developing robust CAD meeting the aforementioned demand is the challenge. These days using a pre-trained model is in trend, which can surpass these limitations.

## 6. Skin Cancer

The diagnosis of melanoma skin cancer illnesses has been conventionally detected by manual analysis and visual examination. These visual examination methods and analyses of skin lesion scans by expert dermatologists are lengthy, complicated, biased, expensive [115]. Melanoma skin cancer is a lethal illness that leads to death when not diagnosed in time. Traditionally, skin cancer was detected based on hand-crafted feature techniques that restrict the high performance of CAD approaches. Current improvements in deep learning methods in medical image analysis and computer vision have led to excessive development of CAD and detection schemes to recognize fatal cancerous skin infections [116].

DL systems automatically extract essential features and have the additional benefit of extracting features directly from the un-processed image. Recently, DCNNs achieved substantial performance in medical imaging [117] and the segmentation of skin lesion images. MAR Ratul et al. [118] proposed an automated CAD system using four CNN-based networks, namely VGG-16, VGG-19, MobileNet, and InceptionV3, to identify malignant skin lesions. The developed architectures were evaluated on the HAM10000 dataset and attained 87.42% for VGG-16, 85.02% for VGG-19, 88.22% for MobileNet, and 89.81% for InceptionV3. In another report, an improved U-Net approach named skinNet was applied using dilated convolutions in the final layer of the encoder section. Its shortcoming includes skin lesion image feature lost by the U-Net shortcut skip connection; thus, a weaker decoder branch in recuperating feature vectors [119]. Esteva et al. [120] implemented a CNN model, trained from scratch, applying dermoscopic images for evaluation that indicated the promising results of the designed CNN network. However, to train an entire architecture from scratch to identify skin lesion cancer utilizing small datasets is usually not realistic. Thus, most scientists typically use fine-tuned methods or pre-trained algorithms through transfer learning.

**Figure 5:**
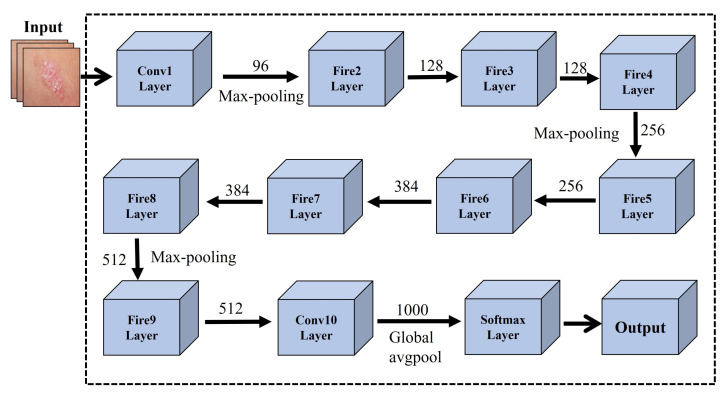
CNN-based SqueezeNet architecture for skin cancer detection.

In another investigation [121], researchers developed a pre-trained architecture using an ensemble of deep learning models including EfficientNets, SENet, and ResNeXt WSL to address skin lesion classification. To attain state-of-the-art results, ISIC 2019 dataset was employed on the proposed model, and certain scaling rules were applied, making it flexible for each image dimension. In another study, [122] developed an effective CNN-based transfer learning SqueezeNet model for Mobile-based skin lesions classification. The networks were trained and tested on 1856 images. The squeezeNet model achieved state-of-the-art results in terms of overall accuracy of 97.21%, sensitivity 94.42%, and specificity 98.14%. CNN-based SqueezeNet architecture is presented in Figure 5.

A fully convolutional residual network (FCRN) based CAD system was suggested by [123], by employing an index calculation unit to refine final results. The mean possibilities of each map are applied for the classification of melanoma skin lesions. Dash et al. [124] introduced an improved U-Net approach called PsLSNet to extract spatial information and used 29 hidden layers of CNN. A novel CNN-based segmentation system was introduced without any preprocessing step [125]. The system learns full resolution features using CNNs. The algorithm was tested on dual datasets called PH2 and ISBI 2017, and the achieved results were compared with other methods, for instance, FCN, SegNet, and U-Net. Although DL-based learning algorithms attain outstanding acknowledgment achievements in the medical imaging domain, more investigation is still needed to improve the performance of CAD systems. The reported CAD systems can never replace doctors but can only assist doctors in the planning of skin cancer diagnosis. Table 5 shows the state-of-the-art CAD approaches of skin cancer.

**Figure 6:**
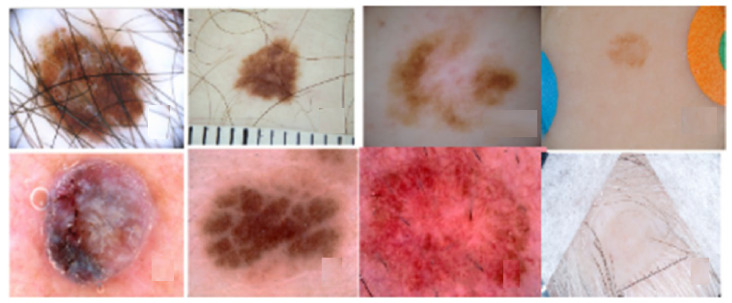
Variation in skin lesion [139].

The systematic review analysis reveals the fact that skin lesion diagnosis is undoubtedly a complex task. It might be attributed to the tremendous variation in image type as well as human skin color and appearance, as illustrated in Figure 6. From the analytical analysis of skin cancer literature data, we determined that ensemble methods with advanced architectures including Resnext, DenseNet121, PsLSNet, InceptionResNetV2, SqueezeNet architecture, Xception, FRCN, ResNet-50 based CNN appears to perform tremendously well. As is assessed from the literature that the utilization of appropriate pre-processing techniques like grab-cut can overcome the skin cancer image analysis challenge. Because deep learning models produce far better classification results when the well-pre-processed images are used as the input.

## 7. Discussion

### 7.1. Primary Observations

**Figure 7:**
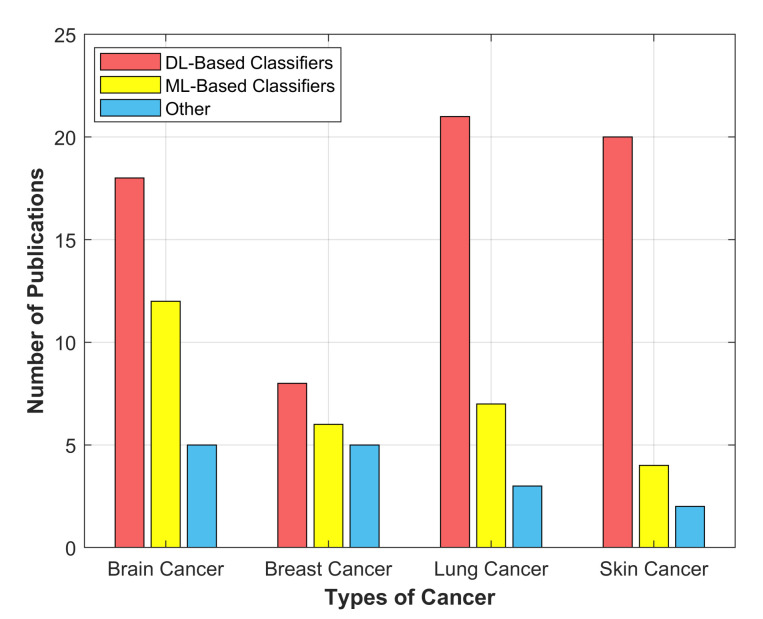
Distribution of publications between different DL-based, ML-based and other classifiers for brain, breast, lung and skin cancer detection.

In this manuscript, we extensively analyze manuscripts published between the years 2016–2021. The comprehensive analysis is depicted in the form of a column graph, as illustrated in Figure 7. One can see that researchers employed more DL-based supervised classifiers (NN, CNN, U-Net, VGG-16, ResNet-50) as compared with ML-based (SVM, DT, RF, K-means) and other classifiers for the detection of various cancers. Deep learning-based unsupervised methods are not considered extensively.

Compared to traditional ML approaches, DL-based CAD systems have been practiced significantly in many applications. In the last few years, it has been found that most researchers have imitated a robust trend towards the development of DL, particularly CNN-based CAD systems. CNN has proven itself a better choice in the medical image analysis field since its emergence in 1989 [140] owing to accurate analysis and time-saving features. Moreover, it is the key solution for many learning problems including feature extraction and object recognition. Summarizing all, CNN has marked great success for detection, classification, and segmentation of multi-organ body cancer attributed to the well-organization and the construction of the layered structure, elements employed in each consecutive layer, and so forth [141,142]. The evident benefit of DL over traditional ML techniques is its less dependency on feature extraction methods as it can automatically develop related features by its hidden layers, that is, convolution and max-pooling [143,144]. Furthermore, DL empowers to directly extract features from input medical image data through a back-propagation operation, which automatically modifies each layer’s weights as stated parameters of supervised learning produced by validated data [144]. In contrast, the execution of a conventional CAD system usually demands regular human participation and constant engineering to confirm its smooth process. CNN-based models can increase the global detection rate of different cancer medical images whenever the inaccurate output is notified through repetitive self-learning skills. It is worth observing that additional repetitions occur between the learning phases; thus, the network’s superior is its sensitivity. Moreover, as CNN extracts and learns features from input images directly thereby, a tumor segmentation step is unnecessary. Nevertheless, in conventional approaches, the segmentation of tumors for multi-organ cancer images is essential for learning appropriate features. Therefore, essential details might have vanished mainly when a wrong segmentation appears [145]. Table 2, Table 3, Table 4 and Table 5 clearly depict determined that the CNN-based techniques [62,67,83,84,107,133] for different body organs-based CAD systems achieved efficient and state-of-the-art performances for the detection, segmentation, and classification using multi-modalities medical images. Additionally, we have highlighted a few loopholes in DL-based approaches discussed in the next section and suggested some recommendations, which can bridge the gap between research studies and early cancer diagnosis.

### 7.2. Open Research Challenges, Possible Solutions and Future Prospects

DL-based methods have significantly enhanced segmentation accuracy, obliged to their ability to handle complicated tasks. Firstly, DL-based methodologies still have not gained full acknowledgment among pathologists for everyday clinical exercise. One of the primary causes might be the lack of standardized medical image acquisition protocols. The unification of the acquisition protocols could minimize it. Secondly, deep learning algorithms usually need massive annotated medical images by expert radiologists to accomplish the training task. Gathering such an enormous annotated dataset of medical images is often a challenging task. Even performing the labeling of a new dataset through experienced experts will also be very time-consuming and costly. Many techniques have been commonly applied to conquer the scarcity of annotated data. Thirdly, the researchers who worked on machine learning algorithms have limited knowledge about the radiology of medical images that is also a barrier in achieving state-of-the-art performance. However, we have discussed other problems and their possible solutions below. This study also provides some recommendations, future directions, and a viewpoint for novice researchers to excel in the medical image analysis domain:

- The most generally implemented technique to extend the range of the training dataset is named data augmentation. It is the application in which different offline changes are done, including affine transformation, cropping, flip, rotation, padding, saturation to the examples [146], and colour augmentation [147];
- Transfer learning from the popular networks [86] employed in the same field or even another area is considered another solution to surpass limited data. It has been established that transfer learning by pre-trained networks produced superior results even when the source and target networks are not the same, transferring the weights of different tasks [148];
- The morphological variation in the cancerous cell is one of the significant issues in medical cancer image detection. The cancerous organ/lesion may differ significantly in dimension, outline, and position from patient to patient [149]. Using deeper architectures can be an effective solution to this issue, as reported in [115]. The unclear border with an imperfect contrast among targeting organs and the nearby tissues in tumor images is an inherent challenge typically produced via attenuation coefficient [150,151]. The use of multi-modality-based methods can solve this issue [152,153];
- The computational complexity of the network is another challenge in DL-based techniques, owing to variability in image dimensions, network construction, or the heavily over-parameterized networks. To evade the powerful GPU hardware constraint and accelerate the segmentation task, one can decrease the number of hidden layers or parameters of the proposed network and emphasize algorithms that artificially generate training data for example GAN [154,155] rather than altering the network;
- The appearance of mostly AI-based architectures seems like a black box. Thus, researchers have no idea about the internal representations of the network and the perfect approach to realize the system completely. Hence, DL approaches are greatly affected by the inherent snags of medical images, that is, noise and illumination. Complete knowledge and understanding of such black box issues in the future would be a revolution in the DL field [156];
- During training time, the ground truth outlines are manually delineated by expert physicians. If manual delineation would be done by a different individual or even the same one at the distinct circumstance, there must be a possibility that the proposed model can be biased and favor expert ground truths as a system error. However, this drawback could be expected to occur in all supervised learning CAD techniques;
- The amalgamation of the robust individual approaches by utilizing their benefits is suitable in further improving the CAD performance. Develop novel CAD systems using hybrid ML-based approaches like SegNet [74], U-Net-Vnet-Fast-R-CNN [157], AgileNet [158] to overcome the complication of overfitting that happens in the training time; this could help in the early diagnosis of multi-organ cancers;
- It is observed that the DL-based unsupervised clustering techniques include; deep auto-encoders, regularized information maximization (RIM), Deep InfoMax (DIM), deep adaptive clustering (DAC), and so forth, have not been engaged widely in comparison with supervised learning techniques [159]. It could avoid the costly training process. These techniques could also be employed to improve the performance of CAD systems in the medical imaging domain.

## 8. Conclusions

This survey critically analyzes four-organ cancer (brain, lung, breast, and skin) diagnosis techniques. The various algorithms were analyzed and discussed in terms of their performances, which were published from 2016 to date. However, the main focus is on recent DL-based approaches. This review work reveals the fact that MRI, CT, dermoscopic images, and mammograms are the gold standard for a brain tumor, lung cancer, skin cancer, and breast cancer diagnosis, respectively. From the comparative analysis, it was found that BRAT, LIDC-IDRI, ISIC and PH2, WDSM, and DDSM are widely employed databases for brain, lung, skin, and breast cancer, respectively. This study provides deep knowledge about the significant positive impact of deep learning on medical image analysis and strengthens the knowledge of the trend and the latest techniques over the past few years. Despite extensive research conducted in this domain so far, there still exists room for improvement. This review has elaborated on the open research challenges and their possible solutions point by point. Moreover, the performance evaluation parameters and publicly available standard datasets for each cancer disease have been briefly discussed. This study will prove highly beneficial for the researchers practicing the deep learning-based cancer diagnosis to design the robust CAD architecture that could be helpful for the medical experts as a second opinion. Some useful recommendations would prove fruitful for researchers as well. In the future, further studies will be conducted to analyze other cancer diseases such as liver and stomach cancers and so forth.

## Figures and Tables

**Table 1:** Commonly utilized publicly available databases with different modalities for brain, breast, lung and skin cancer detection.

Database	Modality	Images/Patients	Link to the Source
BRATS2012	MRI-scans	45 Patients	https://www.smir.ch/BRATS/Start2012 accessed on 30 June 2021
BRATS2015	MRI-scans	274 Patients	https://www.smir.ch/BRATS/Start2015 accessed on 30 June 2021
BRATS2017	MRI-scans	285 Patients	https://www.med.upenn.edu/sbia/brats2017/registration.html accessed on 30 June 2021
BrainWeb	MRI-scans	20	http://www.bic.mni.mcgill.ca/brainweb/ accessed on 30 June 2021
Harvard	MRI-scans	13,000 brain MRIs	http://www.med.harvard.edu/aanlib/ accessed on 30 June 2021
Mini-MIAS	Mammograms	322	http://peipa.essex.ac.uk/info/mias.html accessed on 30 June 2021
DDSM	Mammograms	2620 cases	http://www.eng.usf.edu/cvprg/Mammography/Database.html accessed on 30 June 2021
WBCD	Biopsy	683 Patients	https://archive.ics.uci.edu/ml/datasets/Breast+Cancer+Wisconsin+(Diagnostic) accessed on 30 June 2021
LIDC/IDRI	CT-scans	1018 cases	https://wiki.cancerimagingarchive.net/display/Public/LIDC-IDRI accessed on 30 June 2021
ISIC-2016	Dermoscopic	1279	https://challenge.isic-archive.com/data accessed on 30 June 2021
ISIC-2017	Dermoscopic	2750	https://challenge.isic-archive.com/data accessed on 30 June 2021
HAM10000	Dermoscopic	10,015	https://challenge.isic-archive.com/data accessed on 30 June 2021
PH2	Dermoscopic	200	https://www.fc.up.pt/addi/ph2%20database.html accessed on 30 June 2021
SD-198	Clinical	6584	http://xiaopingwu.cn/assets/projects/sd-198/ accessed on 30 June 2021
SD-260	Clinical	20,660	http://xiaopingwu.cn/assets/projects/sd-198/ accessed on 30 June 2021

**Table 2:** Methodologies of brain tumor cancer diagnosis.

Methods	Task Performed	User Intervention	Dataset	Evaluation Matrix (%)	Year	Ref.
PCA+DNN	Segmentation	Fully-automatic	Harvard	SN = 0.97, Acc = 96.9, AUC = 0.98	2017	[4]
GLCM + Logistic regression (LR)	Segmentation	Fully-automatic	Brats15	SN = 0.88, SP = 0.90, Acc = 0.89, AUC = 0.88	2017	[14]
DWT + Genetic algorithms	Detection	Semi-automatic	Private	Acc = 95.6	2016	[53]
BWT + SVM	Detection	Fully-automatic	BrainWeb	SN = 97.7, SP = 94.2, Acc = 96.5	2017	[36]
GLCM + Gabor + DWT + K-means	Detection	Fully-automatic	Brats15	SN = 89.7, SP = 99.9, Acc = 99.8	2017	[54]
CNN	Segmentation	Fully-automatic	Brats13	WT = 0.78, TC = 0.65, ET = 0.75	2016	[55]
DeepMedic	Segmentation	Fully-automatic	Public	WT = 0.86, ET = 0.78, TC = 0.62	2018	[56]
Integration of FCNNs and CRFs	Segmentation	Fully-automatic	Brats15	WT = 0.84, TC = 0.67, ET = 0.62	2018	[57]
SOM + FKM	Segmentation	Fully-automatic	Harvard	Acc = 96.1, SN = 87.1	2016	[58]
CNN	Segmentation	Fully-automatic	TCGA-GBM	Acc = 90.9	2019	[16]
KNN	Segmentation	Fully-automatic	Brats15	SN = 100, SP = 87.7, Acc = 96.6, AUC = 0.98	2020	[59]
Random forest	Segmentation	Fully-automatic	Brats15	SN = 0.84, SP = 0.71, Acc = 0.87	2019	[60]
Watershed, Gamma Contrast stretching	Classification	-	Harvard	Acc = 0.98	2019	[61]
Multi-Scale 3D U-Nets	Segmentation	Fully-automatic	Brats15	SN = 0.86, SP = 0.86, Acc = 0.85	2020	[62]
TumorGAN	Segmentation	Fully-automatic	Brats17	WT = 0.85, TC = 0.79	2020	[63]
SegNet	Segmentation	Fully-automatic	Brats17	WT = 0.85, TC = 0.81, ET = 0.79	2019	[64]
Two-Channel DNN	Classification	Fully-automatic	Brats18	Acc = 93.69	2021	[65]
DCNN	Classification	Fully-automatic	Private	Acc = 99.25	2021	[66]
Convolutional LSTM XNet	Segmentation	Fully-automatic	Brats19	SN = 0.91, SP = 0.98, Acc = 0.99	2021	[67]
BrainSeg-Net	Segmentation	Fully-automatic	Brats18	WT = 0.89, TC = 0.82, ET = 0.77	2021	[52]

**Table 3:** State-of-the-art CAD systems of breast cancer diagnosis.

Methods	Task Performed	User Intervention	Dataset	Evaluation Matrix (%)	Year	Ref.
Morphological threshold	Mass detection	Automatic	Mini-MIAS	Acc = 94.54	2016	[72]
SSL scheme using CNN	Mass detection	Automatic	Private	Acc = 0.82	2017	[82]
DL	Classification	Automatic	Private	Acc = 93.4, SN = 88.6, SP = 97.1	2016	[83]
CNN	Classification	Automatic	DDSM	Acc = 98.90	2018	[84]
DCNN	Lesions classification	Automatic	-	Acc = 90, SN = 90, SP = 96	2017	[85]
Attention Dense-U-Net	Segmentation	Automatic	DDSM	Acc = 78.3, SN = 77.8, SP = 84.6	2019	[73]
SegNet and U-Net	Tumor Segmentation	Sami-automatic	Private institute	Acc = 68.88, 76.14	2019	[80]
DCNN-SVM-AlexNet	Cancer detection	Sami-automatic	CBIS-DDSM	Acc = 87.2	2019	[81]
CNN based selective kernel U-Net	Segmentation	Automatic	Medical centers	Dice score = 0.826	2020	[79]
OPTICS clustering	Lesion classification	Automatic	DCE-MRI	Acc = 71.4	2020	[71]
Hybrid transfer learning	Cancer detection	Automatic	DDSM	MVGG + ImageNet = 94.3, MVGG = 89.8	2021	[86]
Hybrid VGG-16 and series network, GDDT	Classification	Automatic	-	VGG-16 = 96.45, GDDT = 95.15	2021	[87]
GNRBA	Breast classification	Automatic	WDBC	Acc = 0.98	2017	[88]

**Table 4:** State-of-the-art CAD systems for lung cancer diagnosis.

Methods	Task Performed	Dataset	Evaluation Matrix (%)	Year	Ref.
SVM algorithm	Segmentation	Private	Acc = 89.5	2016	[100]
3D CNN trained on weakly labeled data	Nodule Detection	SPIE-LUNGx	SN = 80	2016	[101]
DCNN	Lung cancer detection	Kaggle, LUNA16	Acc = 0.75, SN = 0.77, SP = 0.74	2017	[102]
Deep residual networks	Nodule classification	LIDC/IDRI	Acc = 89.9, SN = 91, SP = 88.6	2017	[103]
3D-CNN	Detection and Classification	Bowl 2017	Acc = 86.6	2017	[104]
Polygon approximation with SVM	Nodule detection	LIDC	Acc = 98.8, SN = 97.7, SP = 96.2	2018	[90]
Deep residual networks	Nodule classification	LIDC-IDRI	Acc = 0.89, SN = 0.91, SP = 0.88	2017	[103]
Deep learning	Nodule detection	LIDC-IDRI	Acc = 0.96, SN = 0.95, SP = 0.97	2020	[105]
Deep reinforcement learning	Nodule detection	LIDC-IDRI	Acc = 0.64, SN = 0.58, SP = 0.55	2018	[106]
3D nodule candidate	Nodule detection	LIDC	Acc = 0.99, SN = 0.98, SP = 0.98	2019	[107]
Optimized Random Forest	Automatic detection	LIDC-IDRI	Acc = 93.1, SN = 94.8, SP = 91.3, FP = 0.086	2020	[91]
CNN	Segments nodules	LIDC	Acc = 89.8, SN = 85.2, SP = 90.6	2020	[108]
2D DCNN	Nodule detection	LUNA16	SN = 86.42, FP = 73.4	2019	[98]
Generative adversarial networks with DCNN	Nodule classification	Private	SN = 93.9, SP = 77.8	2020	[109]
Patch-Based CNN	Nodule detection	LIDC-IDRI	SN = 92.8	2019	[110]
SVM	Detection and segmentation	Private	SN = 90.6, SP = 73.6	2021	[111]
VGG-16 based CNN	Classifcation	Massachusetts General Hospital (MGH)	Acc = 68.6, SN = 37.5, SP = 82.9, AUC = 0.70	2021	[112]

**Table 5:** State-of-the-art CAD systems of skin cancer/lesion diagnosis.

Methods	Task Performed	Dataset	Evaluation Matrix (%)	Year	Ref.
K-means clustering and SVM	Skin Lesions Detection	Dermweb	Acc = 95.4, SN = 96.8, SP = 89.3	2016	[126]
CNN and SVM	Melanoma classification	DERMIS	Acc = 93.7, SN = 87.5, SP = 100	2016	[127]
CNN	Melanoma lesion segmentation	Dermquest	Acc = 98.5, SN = 95.0, SP = 98.9	2016	[128]
SVM Framework	Melanoma Detection	Public	Acc = 97.32, SN = 98.21, SP = 96.43	2017	[129]
CNNs	Classification	Clinical Images	Acc = 72.0	2017	[120]
Encoder-Decoder with DeepLab and PSPNet	Skin lesion segmentation	ISIC 2018	Acc = 94.2, SN = 90.6, SP = 96.3, Dice = 89.8	2018	[130]
Ensemble Classifiers	Classification	ISIC 2018	Acc = 97.4, SN = 74.7, SP = 95.1, Dice = 97.4	2018	[131]
Fine-tuned neural neworks	Classification	ISIC 2018	Acc = 97.4, SN = 75.7, SP = 95.9, Dice = 97.2	2018	[132]
Deeep Supervised Multi-Scale Network	Skin Cancer Segmentation	ISBI 2017 and PH2	Acc = 94.3, SN = 85.9, Dice = 87.5	2019	[133]
SVM	Skin lesion classification	ISBI 2016	Dice = 77.5, Acc = 85.1	2019	[134]
Neural Networks	Melanoma detection	PH2	Acc = 0.81, SN = 0.72, SP = 0.89	2019	[135]
Full resolution convolutional network (FRCN)	Segmentation	ISBI 2017 and PH2	Acc = 94	2018	[125]
CNN	Detection and Categorization	DermIS	Acc = 95, SN = 93.3	2020	[136]
Deep Learning	Segmentation and classification	MyLab Pathology	Acc = 97.9	2021	[137]
ResNet-50 based CNN and Naive Bayes classifier	Skin lesion classification	Ph2, ISBI2016, and HAM1000	Acc = 95.40, 91.1, 85.50	2021	[138]
